# Anesthetic Considerations in Patients With Achondroplasia

**DOI:** 10.7759/cureus.15832

**Published:** 2021-06-22

**Authors:** Joon-Hyung Kim, Bianca C Woodruff, Michael Girshin

**Affiliations:** 1 Department of Anesthesiology, Westchester Medical Center, Valhalla, USA; 2 Department of Anesthesiology, Metropolitan Hospital Center, New York, USA

**Keywords:** achondroplasia, anesthetic management, dwarfism, preoperative evaluation, spinal anesthesia

## Abstract

Anesthetic management of achondroplastic patients warrants special anatomical and physiological considerations due to significant variations in the airway as well as the spine in regional techniques. In this report, we present the case of a 30-year-old morbidly obese male with achondroplasia, end-stage renal disease (ESRD) on hemodialysis, and renal osteodystrophy, who was scheduled for incision and drainage of a rectal abscess. Preoperative evaluation revealed Mallampati IV airway with a short neck and a scoliotic spine with possible atlantoaxial instability.

## Introduction

Achondroplasia is the most common cause of short-limbed, disproportionate dwarfism, and it has an estimated incidence rate of 1/20,000-1/30,000 live births [1]; it is characterized by macrocephaly and long-bone shortening of proximal upper and lower extremities [2]. Comorbidities that may complicate anesthesia include sleep apnea, obesity, and spinal stenosis. Anesthetic management in these patients warrants special considerations due to the significant variability of airway and spinal anatomy. In this report, we discuss the case of a patient with achondroplasia and perirectal abscess.

## Case presentation

**Figure 1:**
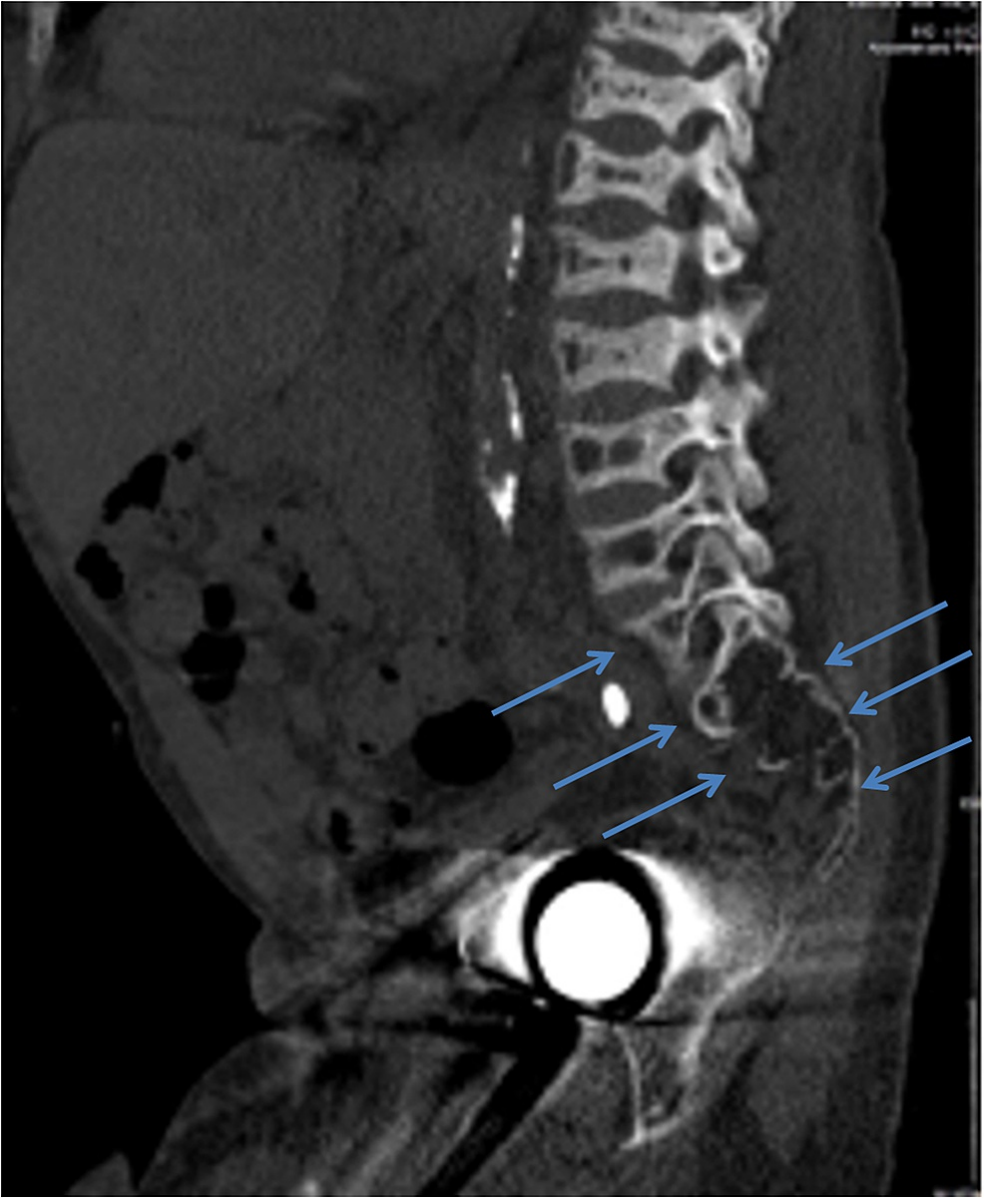
CT of the lumbar spine with marked height loss of lumbar vertebral bodies (arrows) CT: computed tomography

A 30-year-old morbidly obese male with achondroplasia and past medical history of hypertension, end-stage renal disease (ESRD) on hemodialysis after a failed kidney transplant, seizure disorder, and bilateral hip replacement was brought to the operating room for emergent incision and drainage of a right-sided perirectal abscess. Preoperative evaluation revealed a Mallampati IV airway with a short neck, small maxillary overbite, and mild scoliotic cervical spine suggestive of atlantoaxial instability. Lumbar spine CT showed shortening of lumbar vertebral bodies, rugger-jersey vertebral endplate sclerosis consistent with renal osteodystrophy, and a champagne glass-shaped pelvis (Figure 1).

After reviewing anesthetic options and risks, the patient agreed to undergo regional anesthesia. Following ultrasound visualization of the L4-5 interlaminar space while the patient was placed in a lateral decubitus position, the skin was prepared with chlorhexidine-alcohol, and 3 cc of 1% lidocaine was injected subcutaneously. A 25-gauge spinal needle was inserted under ultrasound via paramedian approach for the intrathecal injection of 1 mL of 0.75% bupivacaine hyperbaric and 25 mcg of fentanyl. The patient, placed in a prone position, maintained spontaneous ventilation via face mask throughout the uneventful procedure.

## Discussion

Anesthetic challenges in patients with achondroplasia include potentially difficult airways, often complicated by sleep apnea due to obesity, altered respiratory mechanics, and difficult neuraxial access with the unpredictable spread of local anesthetics. Restrictive lung disease and pulmonary hypertension can develop from chronic hypoxemia or hypercarbia secondary to thoracic scoliosis, airway obstruction, or sleep apnea. Preoperative arterial blood gas may be warranted in these cases.

Neuraxial access can be difficult due to kyphoscoliosis and narrow epidural space. The spine in patients with achondroplasia has several unique anatomical features, such as hypertrophy of superior and inferior articular facets, short and thickened pedicles of the vertebral body from premature fusion with scalloping on the posterior surface, prominent bulging of intervertebral discs, spinal canal tapering caudally from decreases in the interpedicular distance in the lumbar region (as opposed to widening caudally in normal anatomy), and thin dura [3]. These mechanical features can lead to spinal stenosis and nerve root compromise. In patients who had previously undergone lumbar decompression surgeries, scar tissue and altered anatomy can further complicate neuraxial techniques. Because of these expected anatomical challenges, we utilized ultrasound to facilitate access to the intrathecal space. Determining the dose and volume of local anesthetic is further complicated by accentuated lumbar lordosis, spinal stenosis, engorged epidural veins, and narrow epidural and intrathecal spaces, which can result in the unpredictable spread, iatrogenic dural puncture, difficult catheter placement, air embolism, or a high spinal/epidural block [3,4].

There are no guidelines for the selection of the optimal dose of local anesthetics in achondroplastic patients. However, several case reports of achondroplastic patients who underwent spinal anesthesia have been reported in the literature with reduced doses of local anesthetics (Table 1) [4-10].

**Table 1:** Studies describing spinal anesthesia with different optimal doses of local anesthetics in achondroplastic patients C/S: cesarean section

Author	Country	Patient height/weight	Procedure	Spinal dose
Ravenscroft et al. (1998) [4]	South Africa	Not reported	Emergent C/S	1.3 mL 0.5% hyperbaric bupivacaine with 10 mcg fentanyl (total 1.5 mL)
DeRenzo et al. (2005) [5]	USA	124 cm/46 kg	Urgent C/S	Hyperbaric bupivacaine 10 mg with morphine 0.2 mg at L3-4
Mitra et al. (2007) [6]	India	109 cm/45 kg	Emergent C/S	1 mL of 0.5% heavy bupivacaine with fentanyl 10 mcg at L3-4
Bakhshi et al. (2011) [7]	India	134 cm/45 kg	Femur surgery	0.5% hyperbaric bupivacaine 7.5 mg (1.5 mL) at L3-4
Wight et al. (2013) [8]	UK	130 cm/52 kg	Elective C/S	0.5% hyperbaric bupivacaine 5.5 mg with diamorphine 300 mcg (total 1.4 mL)
Inan et al. (2015) [9]	Turkey	148 cm/58 kg	Emergent C/S	Hyperbaric bupivacaine 7.5 mg with morphine 75 mcg, fentanyl 5 mcg (total 2.2 mL) at L3-4
		147 cm/66 kg	Elective C/S	Hyperbaric bupivacaine 7.5 mg with morphine 75 mcg, fentanyl 5 mcg (total 2.2 mL) at L3-4
Lange et al. (2016) [10]	USA	122 cm/33 kg	Elective C/S	0.75% hyperbaric bupivacaine 8.25 mg with morphine 100 mcg, fentanyl 25 mcg
		119 cm/48 kg	Elective C/S	0.75% hyperbaric bupivacaine 6.75 mg with fentanyl 25 mcg

Since the volume and dose needed for an adequate surgical block are difficult to determine as a result of the short stature of the patients, some clinicians advocate for a titratable technique such as epidural or combined spinal-epidural, rather than a single-shot spinal injection [9]. In our case, however, the patient presented to the operating room for a relatively brief operative procedure, and spinal injection was chosen over epidural catheter placement.

If general anesthesia is chosen for these patients, airway challenges during mask ventilation and tracheal intubation should be expected given the characteristic features such as large tongue, narrowed oropharyngeal airway, limited neck extension, and atlantoaxial instability with the risk of cervicomedullary compression [2]. Several facial features resulting from abnormal bone growth, such as a large protruding forehead, short maxilla, large mandible, depressed nasal bridge, and macroglossia, may present challenges during mask ventilation. Furthermore, in achondroplastic patients with atlantoaxial instability, foramen magnum stenosis or cervical kyphoscoliosis, and extension of the neck may need to be severely limited or avoided to prevent cervicomedullary compression, and this may limit visualizing the larynx for tracheal intubation.

Given the risk for brainstem compression, preoperative screening for central obstructive apnea and cervicomedullary compression is critical for the prevention of sudden death from central respiratory failure [11]. In a prospective series of achondroplastic infants, radiographic evidence of craniocervical stenosis was present in 58% of patients and cervicomedullary compression in 35% [12]. Similarly, a 7.5% risk of sudden death in the first year of life was observed in a retrospective study of achondroplastic infants [13]. Presenting signs of cervicomedullary compression include upper or lower extremity paresis, apnea, cyanosis, hyperreflexia, hypertonia, abnormal plantar response, or delay in milestones versus other achondroplastic infants. Awake fiberoptic intubation may be the safest method of securing the airway in these patients if general anesthesia is needed but is not feasible due to a lack of patient cooperation, anxiety, and anatomical challenges secondary to smaller airways. As a result, in-line stabilization with video laryngoscopy may be a good alternative [14].

In a radiographic study of children with achondroplasia, neck flexion resulted in increased intracranial pressure (ICP) due to venous outflow obstruction and blockage of cerebrospinal fluid (CSF) outflow, despite having normal MRI findings in the neutral position, which may present as contraindications for spinal anesthesia [15]. As a result, many clinicians recommend obtaining MRI flow studies of the CSF at the foramen magnum. Respiratory evaluation includes polysomnography, chest radiograph, and testing of blood pH, electrocardiogram, and echocardiogram for the detection of chronic oxygen deprivation during sleep. Hydrocephalus can arise from intracranial venous outflow obstruction and decreased CSF absorption [3]. Neurological findings such as hydrocephalus and increased ICP may present as contraindications for spinal anesthesia, and a thorough neurological exam must be undertaken during the preoperative anesthetic evaluation of achondroplastic patients.

## Conclusions

This case illustrates the successful use of ultrasound-guided spinal anesthesia in an achondroplastic patient without neurologic sequelae. Managing the anesthesia with a spinal block eliminated the need for intubation, reduced the amount of opioids administered, and reduced the risk of airway obstruction in the postoperative period. Understanding the anatomical features associated with achondroplasia is critical for avoiding potential problems with airway management and neuraxial techniques.
